# Supplementary material for: In the Search of Marine Pestiviruses: First Case of Phocoena Pestivirus in a Belt Sea Harbour Porpoise
Source: Viruses. 2022 Jan 17;14(1):161. doi: 10.3390/v14010161 (PMC8780987; doi:10.3390/v14010161)
Supplement: Supplementary file 1 [file viruses-14-00161-s001.zip › Supplementary material/Porpoise pestivirus supplementary material 20220114.pdf]

Supplementary Figure S1

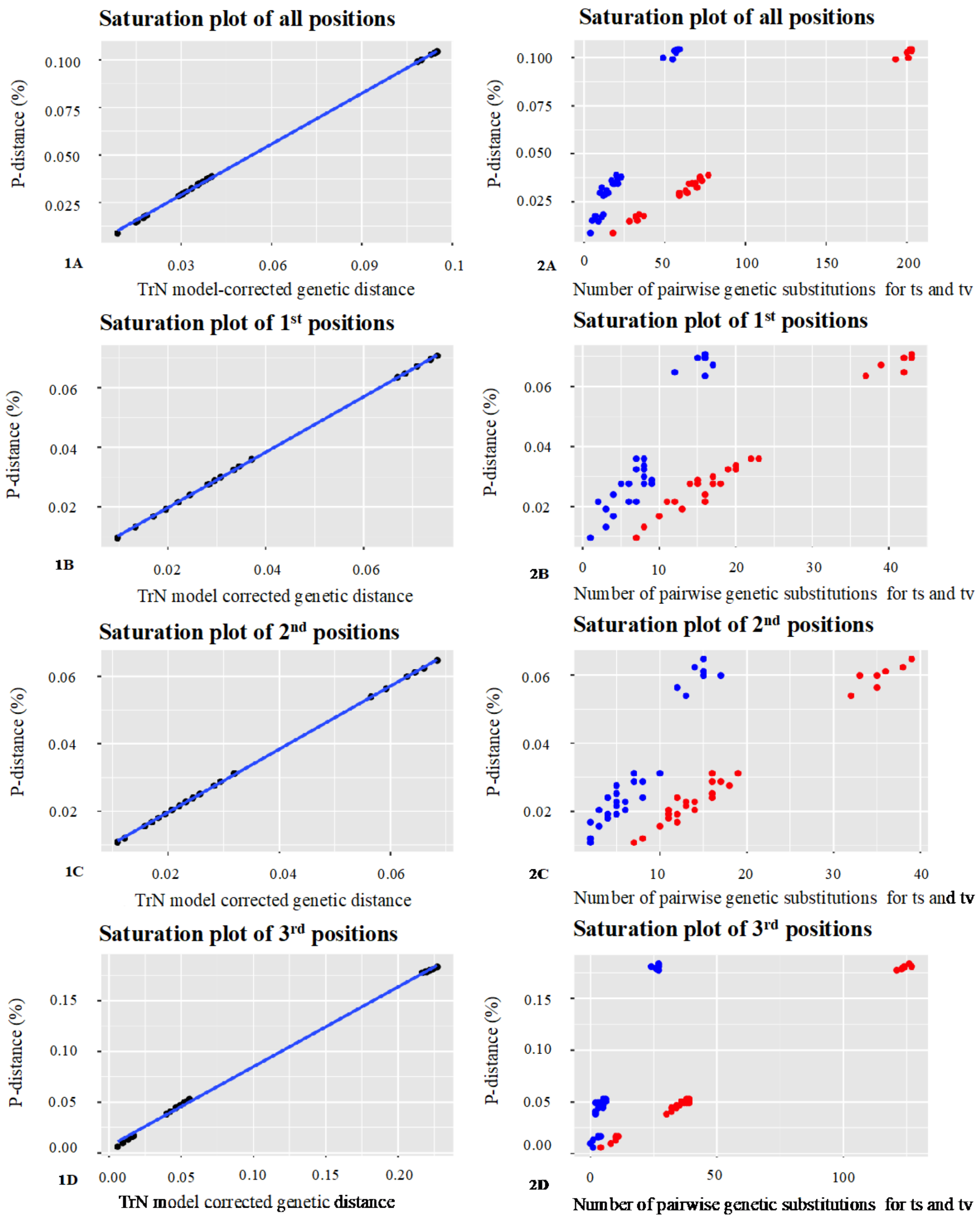

Supplementary Figure S1. Two different substitution saturation plots for the PhoPeV alignment covering all positions, 1st, 2nd and 3rd positions. 1) Plots of uncorrelated pairwise distances vs TrN model-corrected genetic distances (Philippe *et al.*, 1994), where a curved line indicates substitution saturation and a linear line indicates no substitution saturation. 2) Plots of transitions (ts = red) and transversions (tv = blue) (Lydeard and Roe, 1997; Knudsen, et al., 2019), where evidence of

substitution saturation has been found if the red and blue lines cross. In this case no evidence of substitution saturation was detected in either of the plots.

Supplementary Figure S2

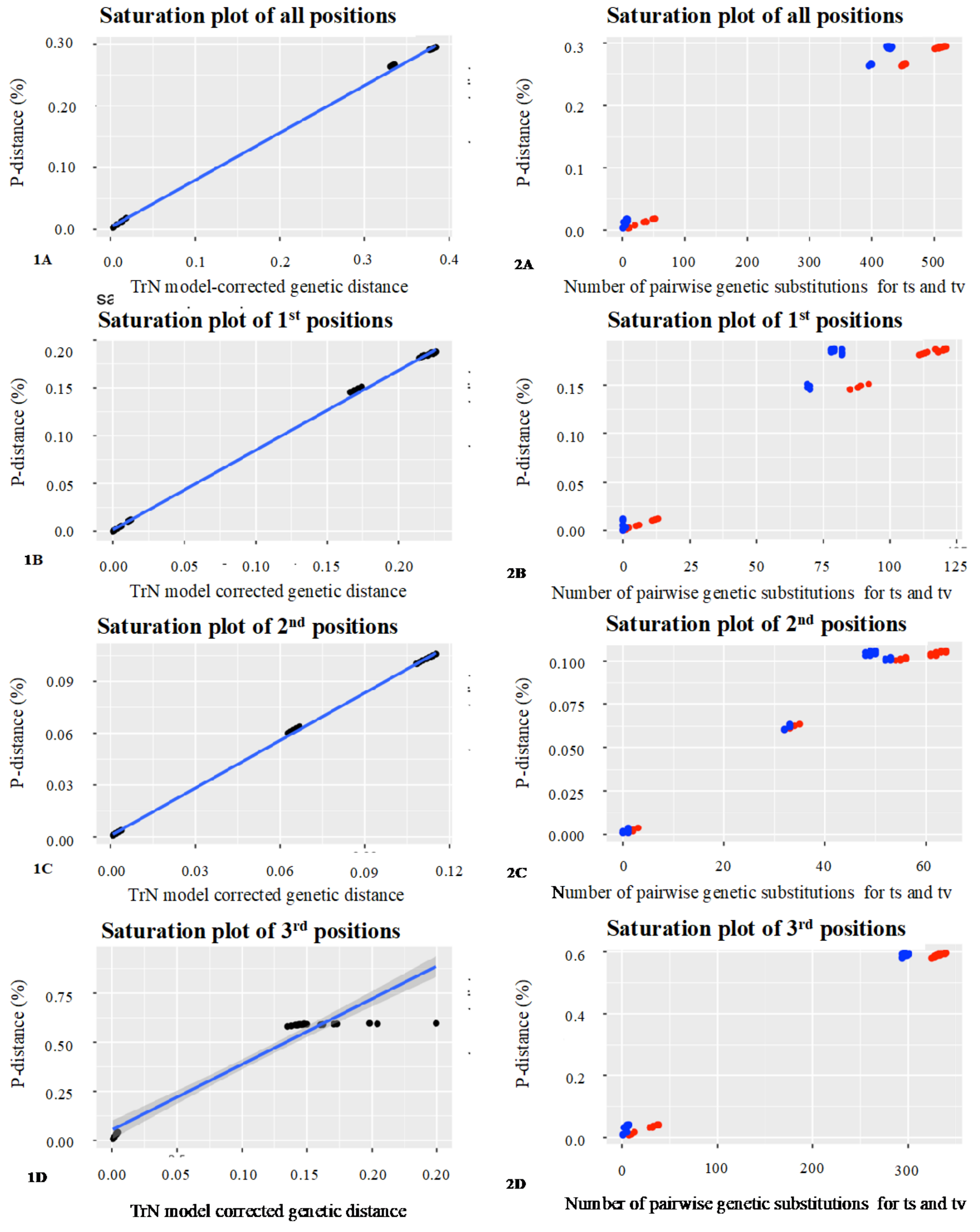

Supplementary Figure S2. Two different substitution saturation plots for the PhoPeV, BuPV, LindaV alignment covering the 1st, 2nd and 3rd positions of conserved region 2 and 3. 1) Plots of uncorrelated pairwise distances vs TrN model-corrected genetic distances (Philippe *et al.*, 1994), where a curved line indicates substitution saturation and a linear line indicates no substitution saturation. 2) Plots of transitions (ts = red) and transversions (tv = blue) (Lydeard and Roe, 1997;

Knudsen, et al., 2019), where evidence of substitution saturation has been found if the red and blue lines cross. In this case evidence of substitution saturation was detected on the 3rd codon position.

Supplementary Table S1. A complete list of the harbour porpoises, harbour seals, grey seals and ringed seals screened throughout this study. Additional sample information such as collection date, sex, age, collection site and sample type are listed when possible. (-) indicates that there is no information. The sample type is indicated by IL = Individual lung tissue extraction, PL = Pooled lung tissue extractions from 3-5 individuals and PMO = Pooled mixed organ extractions from the same individual. * = Samples which were screened separately possibly as pooled samples.

Sample number	Species	Collection date	Sex	Age	Country	Area	Extraction
36267	Harbour porpoise	2007-04-10	Male	Immature	Denmark	Zealand	PL
27238	Harbour porpoise	2007-05-22	Male	Adult	Denmark	Kattegat	IL
35091	Harbour porpoise	2010-01-05	Male	NA	Denmark	Danish straits	PL
35095	Harbour porpoise	2010-03-09	Male	Immature	Denmark	Kattegat	IL
35096	Harbour porpoise	2010-03-15	Female	NA	Denmark	The Sound	PL
35097	Harbour porpoise	2010-03-15	Female	NA	Denmark	The Sound	PL
35098	Harbour porpoise	2010-04-07	Male	NA	Denmark	Kattegat	PL
43703	Harbour porpoise	2010-04-15	Male	NA	Denmark	The Sound	PL
35099	Harbour porpoise	2010-04-22	Male	NA	Denmark	The Sound	PL
35100	Harbour porpoise	2010-04-22	Male	NA	Denmark	The Sound	PL
43702	Harbour porpoise	2010-04-29	Female	NA	Denmark	The Sound	PL
43705	Harbour porpoise	2010-05-02	Male	NA	Denmark	Kattegat	PL
43704	Harbour porpoise	2010-05-13	Male	NA	Denmark	Danish straits	PL
43706	Harbour porpoise	2010-05-28	Male	NA	Denmark	Kattegat	PL
43707	Harbour porpoise	2010-06-03	Female	NA	Denmark	Kattegat	IL
43708	Harbour porpoise	2010-06-04	Male	Immature	Denmark	The Sound	IL
43709	Harbour porpoise	2010-06-04	Male	Immature	Denmark	The Sound	IL
43710	Harbour porpoise	2010-06-08	Male	Immature	Denmark	The Sound	IL
43711	Harbour porpoise	2010-06-19	Female	NA	Denmark	Kattegat	IL
43262	Harbour porpoise	2010-07-14	Male	NA	Denmark	The Sound	PL
43713	Harbour porpoise	2010-07-22	Female	NA	Denmark	The Sound	IL
43721	Harbour porpoise	2010-08-05	Female	NA	Denmark	The Sound	IL
43715	Harbour porpoise	2010-12-04	Male	NA	Denmark	Skagerrak	IL
43720	Harbour porpoise	2011-02-04	Female	NA	Denmark	Zealand	IL
43722	Harbour porpoise	2011-07-02	Female	Adult	Denmark	Kattegat	IL
43717	Harbour porpoise	2011-08-05	Female	NA	Denmark	Danish Straits	IL
43726	Harbour porpoise	2012-07-02	Female	NA	Denmark	North Sea	IL
50735	Harbour porpoise	2015-09-10	Male	NA	Denmark	The Sound	IL
60302	Harbour porpoise	2017-09-28	Female	0 years	Denmark	Danish straits	IL
A2018/05383	Harbour porpoise	2017-11-20	Male	Adult	Sweden	The Sound	IL
A2018/05285	Harbour porpoise	2018-03-14	Female	Subadult	Sweden	The Sound	IL
57027	Harbour porpoise	2018-04-05	Male	NA	Denmark	Danish straits	IL
A2018/05286	Harbour porpoise	2018-04-08	Male	Subadult	Sweden	The Sound	IL
60303	Harbour porpoise	2018-04-19	Mae	NA	Denmark	Danish straits	IL
A2018/05284	Harbour porpoise	2018-12-18	Male	Adult	Sweden	Kattegat (Skåne)	IL
24484	Harbour porpoise	2019-09-21	Male	Juvenile	Germany	Danish Straits	PMO
24476	Harbour porpoise	2019-10-21	Male	Juvenile	Germany	Danish Straits	PMO
24477	Harbour porpoise	2019-11-05	Female	Juvenile	Germany	Danish Straits	PMO

Sample number	Species	Collection date	Sex	Age	Country	Area	Extraction
23288	Harbour porpoise	2018-08-01	Male	NA	Germany	Danish Straits	IL
24263	Harbour porpoise	2018-09-01	Female	Neonate	Denmark	Danish Straits	IL
23616	Harbour porpoise	2018-12-01	Male	NA	Germany	Danish Straits	IL
23817	Harbour porpoise	2019-02-25	Female	NA	Germany	Danish Straits	IL
24247	Harbour porpoise	2019-03-06	Male	Adult	Denmark	The Sound	IL
24250	Harbour porpoise	2019-03-06	Female	Neonate	Denmark	Kattegat	IL
23771	Harbour porpoise	2019-04-16	Male	NA	Germany	North Sea	IL
24256	Harbour porpoise	2019-05-23	Male	Juvenile	Denmark	Kattegat	IL
24257	Harbour porpoise	2019-07-20	Female	Juvenile	Denmark	Kattegat	IL
24239	Harbour porpoise	2019-07-27	Female	Neonate	Denmark	Danish Straits	IL
24023	Harbour porpoise	2019-08-08	Male	NA	Germany	Danish Straits	IL
24144	Harbour porpoise	2019-08-10	Female	Adult	Germany	Danish Straits	IL
24474	Harbour porpoise	2019-08-11	Female	Neonat	Germany	Danish Straits	PMO
24294	Harbour porpoise	2019-08-14	Female	Juvenile	Germany	Danish Straits	PMO
24245	Harbour porpoise	2019-08-22	Male	Neonate	Denmark	Danish Straits	IL
24084	Harbour porpoise	2019-08-24	Male	NA	Germany	Danish Straits	IL
24251	Harbour porpoise	2019-09-05	Male	Adult	Denmark	Bornholm Basin	IL
24468	Harbour porpoise	2019-09-07	Female	Juvenile	Germany	Danish Straits	PMO
24138	Harbour porpoise	2019-09-10	Female	NA	Germany	Danish Straits	IL
24146	Harbour porpoise	2019-09-17	Male	Neonat	Germany	Danish Straits	IL
24148	Harbour porpoise	2019-09-19	Female	Juvenile	Germany	Danish Straits	PMO
24149	Harbour porpoise	2019-09-19	Male	Neonat	Germany	Danish Straits	PMO
24150	Harbour porpoise	2019-09-22	Male	Neonat	Germany	North Sea	IL
24392	Harbour porpoise	2019-09-23	Male	Juvenile	Germany	Danish Straits	PMO
24241	Harbour porpoise	2019-09-27	Male	Juvenile	Denmark	Danish Straits	IL
24391	Harbour porpoise	2019-09-27	Male	Juvenile	Germany	Danish Straits	PMO
24471	Harbour porpoise	2019-09-29	Female	Juvenile	Germany	Danish Straits	PMO
24152	Harbour porpoise	2019-10-02	Male	Juvenile	Germany	Danish Straits	IL
24153	Harbour porpoise	2019-10-02	Male	Neonat	Germany	Danish Straits	IL
24248	Harbour porpoise	2019-10-02	Male	Juvenile	Denmark	Kattegat	IL
24349	Harbour porpoise	2019-10-02	Male	Juvenile	Germany	Danish Straits	PMO
24390	Harbour porpoise	2019-10-04	Female	Juvenile	Germany	Danish Straits	PMO
24470	Harbour porpoise	2019-10-13	Female	Juvenile	Germany	Danish Straits	PMO
24173	Harbour porpoise	2019-10-14	Female	Adult	Germany	Danish Straits	PMO
24472	Harbour porpoise	2019-10-30	Female	Juvenile	Germany	Danish Straits	PMO
24475	Harbour porpoise	2019-11-22	Female	Juvenile	Germany	Danish Straits	PMO
24345	Harbour porpoise	2019-11-24	Female	Adult	Germany	Danish Straits	PMO
24393	Harbour porpoise	2019-12-17	Female	Juvenile	Germany	Danish Straits	PMO
**24341**	Harbour porpoise	NA	NA	NA	Germany	**German Baltic**	PMO

Sample number	Species	Collection date	Sex	Age	Country	Area	Extraction
A2002/05131	Harbour seal	2002-09-07	Male	2	Sweden	Baltic proper	IL
A2004/02986	Harbour seal	2002-07-25	Male	4	Sweden	Skagerrak	IL
A2003/05127	Harbour seal	2003-10-14	Male	?	Sweden	Baltic proper	IL
27194	Harbour seal	2003-05-27	Female	2	Denmark	Danish straits	PL
27193	Harbour seal	2003-08-04	Male	NA	Denmark	Danish straits	PL
34161	Harbour seal	2006-11-15	Female	1-2	Denmark	Danish straits	PL
31610	Harbour seal	2006-09-22	Female	2-3	Denmark	Kattegat	PL
31611	Harbour seal	2006-09-22	Male	0-1	Denmark	Kattegat	PL
37401	Harbour seal	2007-06-21	Female	0	Denmark	Kattegat	PL
37402	Harbour seal	2007-06-21	Male	1-2	Denmark	Kattegat	PL
37403	Harbour seal	2007-06-21	Male	1-2	Denmark	Kattegat	PL
37404	Harbour seal	2007-06-21	Male	> 1	Denmark	Kattegat	PL
37405	Harbour seal	2007-06-21	Female	> 1	Denmark	Kattegat	PL
37406	Harbour seal	2007-06-21	Female	> 1	Denmark	Kattegat	PL
37407	Harbour seal	2007-06-21	Female	> 1	Denmark	Kattegat	PL
37408	Harbour seal	2007-06-21	Female	> 1	Denmark	Kattegat	PL
37409	Harbour seal	2007-06-21	Female	NA	Denmark	Kattegat	PL
35083	Harbour seal	2008-09-30	Female	Adult	Denmark	Danish straits	PL
35082	Harbour seal	2008-06-16	Male	0	Denmark	Kattegat	PL
45408	Harbour seal	2010-08-12	Male	NA	Denmark	Arkona Basin	PL
45401	Harbour seal	2010-11-21	Female	Adult	Denmark	Danish straits	IL
45407	Harbour seal	2010-07-01	Female	Yearling	Denmark	Kattegat	PL
45413	Harbour seal	2011-05-11	Male	Adult	Denmark	Danish straits	PL
45406	Harbour seal	2012-08-12	Female	NA	Denmark	Arkona Basin	PL
45411	Harbour seal	2012-08-12	Male	Adult	Denmark	Arkona Basin	IL
47505	Harbour seal	2012-09-29	Female	2-3	Denmark	Arkona Basin	PL
A2012/05940	Harbour seal	2012-11-13	Male	8	Sweden	Baltic proper	IL
45409	Harbour seal	2012-08-22	Male	Juvenile	Denmark	Danish straits	PL
45410	Harbour seal	2012-05-25	Female	NA	Denmark	The Sound	PL
50714	Harbour seal	2014-11-24	Male	NA	Denmark	Arkona Basin	PL
A2014/05210	Harbour seal	2014-04-01	Male	2	Sweden	Baltic proper	IL
A2015/05106	Harbour seal	2014-09-21	Male	6	Sweden	Baltic proper	IL
51614	Harbour seal	2014-09-13	Male	0	Denmark	Kattegat	PL, IL
50722	Harbour seal	2014-08-29	Female	NA	Denmark	Limfjorden	PL, IL
50723	Harbour seal	2014-08-29	Male	NA	Denmark	Limfjorden	PL, IL
50724	Harbour seal	2014-08-29	Male	NA	Denmark	Limfjorden	PL, IL
50725	Harbour seal	2014-08-29	Female	NA	Denmark	Limfjorden	PL, IL
50730	Harbour seal	2014-02-21	Female	Juvenile	Denmark	North Sea	PL
50727	Harbour seal	2014-04-28	Female	Adult	Denmark	North Sea	PL

Sample number	Species	Collection date	Sex	Age	Country	Area	Extraction
50716	Harbour seal	2014-05-07	Female	Juvenile	Denmark	The Sound	PL
50717	Harbour seal	2014-05-07	Female	Juvenile	Denmark	The Sound	PL
50718	Harbour seal	2014-05-07	Male	Adult	Denmark	The Sound	PL
50720	Harbour seal	2014-08-28	Female	Juvenile	Denmark	The Sound	PL
50721	Harbour seal	2014-08-28	Female	Adult	Denmark	The Sound	PL
A2015/05105	Harbour seal	2015-02-03	Female	3	Sweden	The Sound	IL
M1515	Harbour seal	2015-04-07	Female	Juvenile	Denmark	The Sound	PL
M1516	Harbour seal	2016-01-15	Male	Adult	Denmark	The Sound	PL
A2019/05494	Harbour seal	2018-10-11	Male	NA	Sweden	Bornholm Basin	IL
24232	Harbour seal	2018-08-20	Male	0	Denmark	Danish Straits	IL
24266	Harbour seal	2018-10-29	Female	Adult	Denmark	Danish Straits	IL
23514	Harbour seal	2018-11-01	Male	NA	Germany	Danish Straits	IL
A2019/05259	Harbour seal	2018-11-21	Female	Adult	Sweden	Kattegat	IL
A2019/05257	Harbour seal	2018-12-25	Male	Adult	Sweden	Kattegat	IL
A2019/05134	Harbour seal	2018-10-01	Female	Adult	Sweden	Kattegat	IL
A2019/05136	Harbour seal	2018-10-01	Female	0-1	Sweden	Kattegat	IL
A2019/05138	Harbour seal	2018-10-01	Male	0-1	Sweden	Kattegat	IL
A2019/05131	Harbour seal	2018-10-04	Female	0-1	Sweden	Kattegat	IL
A2019/05132	Harbour seal	2018-10-04	Male	0-1	Sweden	Kattegat	IL
A2019/05130	Harbour seal	2018-10-07	Female	NA	Sweden	Kattegat	IL
A2019/05135	Harbour seal	2018-10-07	Female	0-1	Sweden	Kattegat	IL
23398	Harbour seal	2018-01-13	Female	NA	Germany	North Sea	IL
23697	Harbour seal	2018-03-05	Male	NA	Germany	North Sea	IL
23389	Harbour seal	2018-10-05	Male	NA	Germany	North Sea	IL
A2019/05137	Harbour seal	2018-10-01	NA	NA	Sweden	Skagerrak	IL
A2019/05133	Harbour seal	2018-10-12	Female	Adult	Sweden	Skagerrak	IL
43790	Harbour seal	2018-10-10	Male	Juvenile	Denmark	The Sound	PL
43791	Harbour seal	2018-10-10	NA	NA	Denmark	The Sound	PL
24260	Harbour seal	2019-08-11	Female	0	Denmark	Danish Straits	IL
24246	Harbour seal	2019-08-28	Male	Yearling	Denmark	Danish Straits	IL
24235	Harbour seal	2019-09-18	Female	0	Denmark	Danish Straits	IL
24254	Harbour seal	2019-10-09	Male	Yearling	Denmark	Danish Straits	IL
24259	Harbour seal	2019-10-22	Female	Yearling	Denmark	Danish Straits	IL
24234	Harbour seal	2019-04-28	Male	Yearling	Denmark	Kattegat	IL
24236	Harbour seal	2019-04-28	Female	Yearling	Denmark	Kattegat	IL
23725	Harbour seal	2019-03-10	Female	NA	Germany	North Sea	IL
23966	Harbour seal	2019-07-02	Male	NA	Germany	North Sea	IL
23997	Harbour seal	2019-07-23	Male	NA	Germany	North Sea	IL

Sample number	Species	Collection date	Sex	Age	Country	Area	Extraction
24238	Harbour seal	2019-04-07	Male	Yearling	Denmark	The Sound	IL
24255	Harbour seal	2019-05-15	Female	Adult	Denmark	The Sound	IL
24237	Harbour seal	2019-05-24	Male	0	Denmark	The Sound	IL
24249	Harbour seal	2019-06-03	Male	Yearling	Denmark	The Sound	IL
24253	Harbour seal	2019-06-03	Female	Adult	Denmark	The Sound	IL
24258	Harbour seal	2019-08-19	Female	0	Denmark	The Sound	IL
24267	Harbour seal	2019-08-24	Male	Adult	Denmark	The Sound	IL
23860	Harbour seal	2018-2019	NA	NA	Germany	North Sea	IL

Sample number	Species	Collection date	Sex	Age	Country	Area	Extraction
A2003/05034	Grey seal	2002-12-28	Male	3	Sweden	Arkona Basin	IL
C2002/05362	Grey seal	2002-10-25	Male	14	Sweden	Baltic proper	IL
C2002/05281	Grey seal	2002-05-10	Female	13	Sweden	Bothnian Bay	IL
C2002/05295	Grey seal	2002-11-03	Female	3	Sweden	Bothnian Bay	IL
C2002/05040	Grey seal	2002-04-25	Female	0-1	Sweden	Bothnian Sea	IL
C2002/05114	Grey seal	2002-08-20	Female	0-1	Sweden	Bothnian Sea	IL
A2002/05142	Grey seal	2002-10-21	Female	11	Sweden	Bothnian Sea	IL
A2002/05039	Grey seal	2002-04-03	Male	6	Sweden	Skagerrak	IL
C2003/05014	Grey seal	2003-05-25	Female	5	Sweden	Baltic proper	IL
A2003/05143	Grey seal	2003-11-16	Male	32	Sweden	Baltic proper	IL
C2003/05012	Grey seal	2003-04-22	Male	0-1	Sweden	Bornholm Basin	IL
C2003/05008	Grey seal	2003-05-04	Male	3	Sweden	Bothnian Sea	IL
C2003/05254	Grey seal	2003-07-08	Female	3	Sweden	Bothnian Sea	IL
45404	Grey seal	2010-06-26	Male	Young	Denmark	Kattegat	PL
35093	Grey seal	2010-03-03	Male	Adult	Denmark	The Sound	PL
45412	Grey seal	2012-08-12	Male	-	Denmark	SW Baltic	PL
A2014/05339	Grey seal	2013-04-21	Female	3	Sweden	Baltic proper	IL
A2013/05351	Grey seal	2013-08-07	Male	0	Sweden	Baltic proper	IL
A2013/05455	Grey seal	2013-05-11	Female	0	Sweden	Bothnian Bay	IL
A2014/05497	Grey seal	2013-07-25	Female	5	Sweden	Bothnian Bay	IL
A2014/05492	Grey seal	2013-04-27	Male	17	Sweden	Bothnian Sea	IL
A2014/05484	Grey seal	2013-08-01	Female	6	Sweden	Bothnian Sea	IL
50701	Grey seal	2014-01-15	NA	NA	Denmark	Arkona Basin	PL
50704	Grey seal	2014-11-22	Male	NA	Denmark	Arkona Basin	PL
A2014/05235	Grey seal	2014-04-25	Female	19	Sweden	Baltic proper	IL
A2014/05472	Grey seal	2014-08-25	Female	3	Sweden	Baltic proper	IL
A2015/05299	Grey seal	2014-12-31	Male	2	Sweden	Bornholm Basin	IL
A2015/05300	Grey seal	2014-12-31	Male	2	Sweden	Bornholm Basin	IL
A2014/05503	Grey seal	2014-05-11	Male	8	Sweden	Bothnian Bay	IL
A2017/05800	Grey seal	2014-08-24	Male	10	Sweden	Bothnian Bay	IL
A2014/05514	Grey seal	2014-07-19	Male	4	Sweden	Bothnian Sea	IL
A2015/05080	Grey seal	2014-10-24	Male	2	Sweden	Bothnian Sea	IL
50702	Grey seal	2014-05-02	NA	Pub	Denmark	Danish Straits	PL
50714	Grey seal	2014-04-23	Female	Adult	Denmark	SW Baltic	PL
50728	Grey seal	2014-11-22	Male	Adult	Denmark	The Sound	PL
50726	Grey seal	2014-03-02	Female	Juvenile	Denmark	West coast	PL
A2015/05322	Grey seal	2015-04-28	Male	7	Sweden	Baltic proper	IL
A2015/05496	Grey seal	2015-05-08	Male	5	Sweden	Bornholm Basin	IL
A2015/05544	Grey seal	2015-08-13	Female	8	Sweden	Bornholm Basin	IL

Sample number	Species	Collection date	Sex	Age	Country	Area	Extraction
A2016/05148	Grey seal	2015-11-03	Male	12	Sweden	Bornholm Basin	IL
A2015/05572	Grey seal	2015-05-08	Male	18	Sweden	Bothnian Bay	IL
A2019/05143	Grey seal	2015-04-24	Female	NA	Sweden	Bothnian Sea	IL
A2019/05207	Grey seal	2015-04-24	NA	NA	Sweden	Bothnian Sea	IL
A2019/05144	Grey seal	2015-04-25	Male	NA	Sweden	Bothnian Sea	IL
A2019/05145	Grey seal	2015-04-25	Male	NA	Sweden	Bothnian Sea	IL
A2015/05543	Grey seal	2015-07-15	Female	2	Sweden	Bothnian Sea	IL
A2019/05237	Grey seal	2016-09-19	Female	NA	Sweden	Bothnian Sea	IL
23555	Grey seal	2017-07-12	Female	Yearling	Poland	Bornholm Basin	IL
A2019/05378	Grey seal	2018-09-02	Female	Adult	Sweden	Arkona Basin	IL
A2019/05514	Grey seal	2018-09-02	Male	Adult	Sweden	Arkona Basin	IL
A2019/05564	Grey seal	2018-09-02	Female	Adult	Sweden	Arkona basin	IL
23564	Grey seal	2018-03-16	Male	Adult	Poland	Baltic proper	IL
23566	Grey seal	2018-04-05	Male	Pup	Poland	Baltic proper	IL
23573	Grey seal	2018-06-14	Female	Yearling	Poland	Baltic proper	IL
A2019/05139	Grey seal	2018-08-05	Male	Juvenile	Sweden	Baltic proper	IL
A2019/05142	Grey seal	2018-08-05	Male	Adult	Sweden	Baltic proper	IL
A2019/05243	Grey seal	2018-08-05	Male	Juvenile	Sweden	Baltic proper	IL
A2019/05244	Grey seal	2018-08-05	Male	Juvenile	Sweden	Baltic proper	IL
A2019/05246	Grey seal	2018-08-05	Male	Juvenile	Sweden	Baltic proper	IL
A2019/05248	Grey seal	2018-08-05	Female	Juvenile	Sweden	Baltic proper	IL
A2019/05563	Grey seal	2018-08-05	Male	Juvenile	Sweden	Baltic proper	IL
A2019/05140	Grey seal	2018-08-06	Male	Adult	Sweden	Baltic proper	IL
A2019/05245	Grey seal	2018-08-07	Male	Juvenile	Sweden	Baltic proper	IL
A2019/05247	Grey seal	2018-08-07	Female	Juvenile	Sweden	Baltic proper	IL
A2019/05380	Grey seal	2018-08-29	Male	NA	Sweden	Baltic proper	IL
A2019/05515	Grey seal	2018-08-31	Male	Juvenile	Sweden	Baltic proper	IL
A2019/05382	Grey seal	2018-09-05	Female	NA	Sweden	Baltic proper	IL
A2019/05567	Grey seal	2018-09-13	Male	NA	Sweden	Baltic proper	IL
A2019/05512	Grey seal	2018-09-19	Male	Juvenile	Sweden	Baltic proper	IL
23585	Grey seal	2018-10-25	Male	Yearling	Poland	Baltic proper	IL
A2019/05229	Grey seal	2018-?-?	Female	NA	Sweden	Baltic proper	IL
A2019/05269	Grey seal	2018-?-?	Female	Juvenile	Sweden	Baltic proper	IL
23569	Grey seal	2018-05-01	Male	Yearling	Poland	Bornholm Basin	IL
A2019/05383	Grey seal	2018-08-05	Male	NA	Sweden	Bornholm Basin	IL
A2019/05216	Grey seal	2018-08-29	Female	Adult	Sweden	Bornholm Basin	IL
A2019/05269	Grey seal	2018-?-?	Female	Juvenile	Sweden	Baltic proper	IL
23569	Grey seal	2018-05-01	Male	Yearling	Poland	Bornholm Basin	IL
A2019/05383	Grey seal	2018-08-05	Male	NA	Sweden	Bornholm Basin	IL

Sample number	Species	Collection date	Sex	Age	Country	Area	Extraction
A2019/05216	Grey seal	2018-08-29	Female	Adult	Sweden	Bornholm Basin	IL
A2019/05511	Grey seal	2018-09-07	Female	NA	Sweden	Bornholm Basin	IL
A2019/05211	Grey seal	2018-08-26	Male	Adult	Sweden	Borrnholm Basin	IL
A2019/05242	Grey seal	2018-09-02	NA	NA	Sweden	Bothnian Bay	IL
A2019/05267	Grey seal	2018-09-02	Female	Juvenile	Sweden	Bothnian Bay	IL
A2019/05146	Grey seal	2018-08-02	Female	Juvenile	Sweden	Bothnian Sea	IL
A2019/05226	Grey seal	2018-08-02	Male	Juvenile	Sweden	Bothnian Sea	IL
A2019/05266	Grey seal	2018-08-02	NA	NA	Sweden	Bothnian Sea	IL
A2019/05275	Grey seal	2018-08-03	Male	Subadult	Sweden	Bothnian Sea	IL
A2019/05516	Grey seal	2018-08-03	Female	Adult	Sweden	Bothnian Sea	IL
A2019/05215	Grey seal	2018-08-04	Female	Adult	Sweden	Bothnian Sea	IL
A2019/05230	Grey seal	2018-08-04	Female	Adult	Sweden	Bothnian Sea	IL
A2019/05223	Grey seal	2018-08-10	Male	NA	Sweden	Bothnian Sea	IL
A2019/05141	Grey seal	2018-08-14	Male	Adult	Sweden	Bothnian Sea	IL
A2019/05213	Grey seal	2018-08-14	Female	Adult	Sweden	Bothnian Sea	IL
A2019/05214	Grey seal	2018-08-14	Female	Adult	Sweden	Bothnian Sea	IL
A2019/05222	Grey seal	2018-08-15	Male	Adult	Sweden	Bothnian Sea	IL
A2019/05231	Grey seal	2018-08-15	Female	Juvenile	Sweden	Bothnian Sea	IL
A2019/05238	Grey seal	2018-08-15	Male	NA	Sweden	Bothnian Sea	IL
A2019/05268	Grey seal	2018-08-15	Female	Juvenile	Sweden	Bothnian Sea	IL
A2019/05224	Grey seal	2018-08-20	Male	Adult	Sweden	Bothnian Sea	IL
A2019/05227	Grey seal	2018-08-20	Female	Adult	Sweden	Bothnian Sea	IL
A2019/05208	Grey seal	2018-08-22	Female	Adult	Sweden	Bothnian Sea	IL
A2019/05225	Grey seal	2018-08-22	Male	Adult	Sweden	Bothnian Sea	IL
A2019/05270	Grey seal	2018-08-22	Female	Juvenile	Sweden	Bothnian Sea	IL
A2019/05274	Grey seal	2018-08-22	Female	Adult	Sweden	Bothnian Sea	IL
A2019/05209	Grey seal	2018-08-24	Female	Adult	Sweden	Bothnian Sea	IL
A2019/05228	Grey seal	2018-08-25	Female	Adult	Sweden	Bothnian Sea	IL
A2019/05273	Grey seal	2018-08-25	Female	Adult	Sweden	Bothnian Sea	IL
A2019/05147	Grey seal	2018-09-02	Male	Adult	Sweden	Bothnian Sea	IL
A2019/05241	Grey seal	2018-09-02	Female	Juvenile	Sweden	Bothnian Sea	IL
A2019/05513	Grey seal	2018-09-02	Female	Juvenile	Sweden	Bothnian Sea	IL
A2019/05271	Grey seal	2018-09-04	Female	Adult	Sweden	Bothnian Sea	IL
A2019/05272	Grey seal	2018-09-04	Female	Adult	Sweden	Bothnian Sea	IL
A2019/05375	Grey seal	2018-09-04	Female	Adult	Sweden	Bothnian Sea	IL
A2019/05376	Grey seal	2018-09-04	Female	Adult	Sweden	Bothnian Sea	IL
A2019/05562	Grey seal	2018-09-07	Female	NA	Sweden	Bothnian Sea	IL
A2019/05210	Grey seal	2018-09-13	Male	Adult	Sweden	Bothnian Sea	IL
A2019/05239	Grey seal	2018-10-01	Female	Juvenile	Sweden	Bothnian Sea	IL

Sample number	Species	Collection date	Sex	Age	Country	Area	Extraction
A2019/05212	Grey seal	2018-10-20	Male	NA	Sweden	Bothnian Sea	IL
23287	Grey seal	2018-07-31	Female	Adult	Germany	North Sea	IL
24265	Grey seal	2019-02-05	Female	NA	Denmark	Arkona Basin	IL
24264	Grey seal	2019-10-01	Male	Adult	Denmark	Arkona Basin	IL
A2019/05569	Grey seal	2019-08-03	Male	NA	Sweden	Baltic proper	IL
A2019/05381	Grey seal	2019-08-15	Male	NA	Sweden	Baltic proper	IL
A2019/05566	Grey seal	2019-09-04	Male	NA	Sweden	Baltic proper	IL
A2019/05627	Grey seal	2019-09-16	Female	NA	Sweden	Baltic proper	IL
A2019/05148	Grey seal	2019-10-15	Male	NA	Sweden	Baltic proper	IL
A2019/05570	Grey seal	2019-08-10	Fenale	NA	Sweden	Bothnian Sea	IL
23733	Grey seal	2019-03-23	NA	NA	Germany	North Sea	IL
24145	Grey seal	2019-04-14	Male	NA	Poland	NA	IL

Sample number	Species	Collection date	Sex	Age	Country	Area	Extraction
A2002/05049	Ringed seal	2002-04-29	Female	2	Sweden	Baltic proper	PL
A2002/05132	Ringed seal	2002-09-30	Female	0-1	Sweden	Baltic proper	PL
A2002/05129	Ringed seal	2002-09-22	Male	0-1	Sweden	Bothnian Sea	PL
A2002/05130	Ringed seal	2002-09-24	Male	NA	Sweden	Bothnian Sea	PL
A2003/05071	Ringed seal	2003-06-26	Female	NA	Sweden	Bothnian Bay	PL
A2003/05072	Ringed seal	2003-05-05	Male	0-1	Sweden	Bothnian Sea	PL
A2003/05089	Ringed seal	2003-08-22	Female	NA	Sweden	Bothnian Sea	PL
A2003/05134	Ringed seal	2003-10-13	Male	NA	Sweden	Bothnian Sea	PL
A2006/05406	Ringed seal	2006-10-25	Male	0-1	Sweden	Baltic proper	PL
A2006/05390	Ringed seal	2006-10-11	Female	0-1	Sweden	Bornholm Basin	PL
A2006/05441	Ringed seal	2006-12-13	Male	NA	Sweden	Bothnian Sea	PL
A2007/05299	Ringed seal	2007-09-19	Female	Adult	Sweden	Bothnian Bay	PL
A2008/05113	Ringed seal	2007-05-11	Male	0-1	Sweden	Bothnian Sea	PL
A2007/05223	Ringed seal	2007-06-26	Male	NA	Sweden	Bothnian Sea	PL
A2007/05320	Ringed seal	2007-10-02	Male	NA	Sweden	Bothnian Sea	PL
A2007/05395	Ringed seal	2007-11-05	Female	1	Sweden	Bothnian Sea	PL
A2008/05299	Ringed seal	2008-05-23	Male	12	Sweden	Bothnian Bay	PL
A2008/05330	Ringed seal	2008-05-28	Female	13	Sweden	Bothnian Bay	PL
A2008/05347	Ringed seal	2008-05-29	Female	7	Sweden	Bothnian Bay	PL
A2009/05052	Ringed seal	2008-06-16	Male	3	Sweden	Bothnian Bay	PL
A2008/05197	Ringed seal	2008-04-06	Male	0-1	Sweden	Bothnian Sea	PL
A2008/05394	Ringed seal	2008-05-20	Female	0-1	Sweden	Bothnian Sea	PL
A2008/05429	Ringed seal	2008-08-02	Female	0-1	Sweden	Bothnian Sea	PL
A2014/05193	Ringed seal	2013-04-22	Male	11	Sweden	Baltic proper	PL
A2013/05447	Ringed Seal	2013-08-27	Female	0-1	Sweden	Bothnian Bay	PL
A2018/05190	Ringed seal	2013-11-01	Female	3	Sweden	Bothnian Bay	PL
A2014/05703	Ringed seal	2013-11-02	Male	12	Sweden	Bothnian Bay	PL
A2013/05342	Ringed seal	2013-08-02	Male	14	Sweden	Bothnian Sea	PL
A2014/05144	Ringed seal	2013-11-19	Male	3	Sweden	Bothnian Sea	PL
A2015/05316	Ringed seal	2014-09-28	Male	0-1	Sweden	Baltic proper	PL
A2015/05292	Ringed seal	2014-06-20	Male	0-1	Sweden	Bothnian Bay	PL
A2015/05320	Ringed seal	2014-10-17	Male	1	Sweden	Bothnian Bay	PL
A2014/05650	Ringed seal	2014-11-03	Female	23	Sweden	Bothnian Bay	PL
A2015/05079	Ringed seal	2014-04-02	Male	0-1	Sweden	Bothnian Sea	PL
A2015/05317	Ringed seal	2014-05-26	Male	0-1	Sweden	Bothnian Sea	PL
A2015/05224	Ringed seal	2014-06-28	Female	0-1	Sweden	Bothnian Sea	PL
A2016/05133	Ringed seal	2015-10-09	Female	0-1	Sweden	Baltic proper	PL
A2015/05733	Ringed seal	2015-05-30	Male	29	Sweden	Bothnian Bay	PL

Sample number	Species	Collection date	Sex	Age	Country	Area	Extraction
A2015/05559	Ringed seal	2015-06-22	Female	1	Sweden	Bothnian Bay	PL
A2018/05118	Ringed seal	2015-11-19	Female	10	Sweden	Bothnian Bay	PL
A2018/05095	Ringed seal	2015-12-13	Male	5	Sweden	Bothnian Bay	PL
A2015/05492	Ringed seal	2015-04-15	Female	3	Sweden	Bothnian Sea	PL
A2017/05370	Ringed seal	2015-05-27	Female	0-1	Sweden	Bothnian Sea	PL
A2017/05816	Ringed seal	2017-05-06	Male	18	Sweden	Bothnian Bay	PL
A2018/05280	Ringed seal	2017-08-01	Female	9	Sweden	Bothnian Bay	PL
A2018/05297	Ringed seal	2017-08-21	Male	8	Sweden	Bothnian Bay	PL
A2018/05485	Ringed seal	2017-09-24	Male	5	Sweden	Bothnian Bay	PL
A2018/05153	Ringed seal	2017-10-01	Male	26	Sweden	Bothnian Bay	PL
A2018/05290	Ringed seal	2017-10-05	Female	17	Sweden	Bothnian Bay	PL
A2018/05489	Ringed seal	2017-10-22	Female	11	Sweden	Bothnian Bay	PL
A2018/05281	Ringed seal	2017-10-29	Female	11	Sweden	Bothnian Bay	PL
A2018/05579	Ringed seal	2017-11-05	Female	0-1	Sweden	Bothnian Bay	PL
A2018/05144	Ringed seal	2017-06-03	Male	0-1	Sweden	Bothnian Sea	PL
A2018/05086	Ringed seal	2017-10-20	Female	0-1	Sweden	Bothnian Sea	IL
A2018/05151	Ringed seal	2017-12-18	Male	0-1	Sweden	Bothnian Sea	PL
A2018/05298	Ringed seal	2018-03-15	Male	23	Sweden	Baltic proper	PL
23204	Ringed seal	2018-06-12	Female	NA	Sweden	Bothnian Bay	PL
23205	Ringed seal	2018-06-13	Male	NA	Sweden	Bothnian Bay	PL
23206	Ringed seal	2018-06-14	Male	NA	Sweden	Bothnian Bay	PL
23207	Ringed seal	2018-06-14	Female	NA	Sweden	Bothnian Bay	PL
23208	Ringed seal	2018-06-14	Female	NA	Sweden	Bothnian Bay	PL
A2018/05694	Ringed seal	2018-10-18	Male	NA	Sweden	Bothnian Bay	IL
23319	Ringed seal	2018-09-02	Male	NA	Sweden	Danish straits	IL

Supplementary Table S2. Information on sequences included in the PhoPeV phylogeny.

Sample ID	Species	Host	Find date	Location	Sequence name (genome)	Tissue	Sequence name (genome)	Tissue	Sequence ID (P)	Tissue	Sequence ID (E2)	Tissue
43720	PhoPeV	Harbour porpoise	20110204	Denmark	New sequence	Lung						
NS170385	PhoPeV	Harbour porpoise	201205	Netherlands	MK910227	Lung						
NS170386	PhoPeV	Harbour porpoise	200812	Netherlands	MK910229	Lung	MK910228	Brain				
NS190100	PhoPeV	Harbour porpoise	201409	Netherlands					MK910230	Kidney	MK910234	Kidney
NS190109	PhoPeV	Harbour porpoise	200507	Netherlands					MK910231	Kidney	MK910235	Kidney
NS190022	PhoPeV	Harbour porpoise	200106	Netherlands					MK910232	Kidney	MK910236	Kidney
NS190017	PhoPeV	Harbour porpoise	200404	Netherlands					MK910233	Kidney	MK910237	Kidney

Supplementary Table S3. Information on sequences used in the PhoPeV, BuPV and LindaV analyses.

Sample ID	Genbank	Coverage	Virus	Host	Find date	Location
43720	New sequence	Genome	PhoPeV	Harbour porpoise	2011-02-04	Denmark
NS170385	MK910227	Genome	PhoPeV	Harbour porpoise	2012-05-?	Netherlands
NS170386	MK910229/28	Genome	PhoPeV	Harbour porpoise	2008-12-?	Netherlands
NS190100	MK910234	E2	PhoPeV	Harbour porpoise	2014-09-?	Netherlands
NS190109	MK910235	E2	PhoPeV	Harbour porpoise	2005-07-?	Netherlands
NS190022	MK910236	E2	PhoPeV	Harbour porpoise	2001-06-?	Netherlands
NS190017	MK910237	E2	PhoPeV	Harbour porpoise	2004-04-?	Netherlands
03/2007-9	MH807261	Genome	BuPV	Pig	2003-08-06	Australia
E570/E2/2010	MH807262	Genome	BuPV	Pig	2012-09-29	Australia
H482/E2/2014	MH807263	Genome	BuPV	Pig	2014-01-09	Australia
C843/E2/2003	MH712512	E2	BuPV	Pig	2003-07-21	Australia
E570/E2/2010	MH712513	E2	BuPV	Pig	2010-09-29	Australia
E997/E2/2011	MH712514	E2	BuPV	Pig	2011-08-29	Australia
03/2007-5	MH712515	E2	BuPV	Pig	2003-08-06	Australia
03/2007-9	MH712516	E2	BuPV	Pig	2003-08-06	Australia
E889/E2/2011	MH712517	E2	BuPV	Pig	2011-06-08	Australia
H153/E2/2013	MH712518	E2	BuPV	Pig	2013-07-24	Australia
H151/E2/2013	MH712519	E2	BuPV	Pig	2013-07-24	Australia
H482/E2/2014	MH712520	E2	BuPV	Pig	2014-01-09	Australia
H624/E2/2014	MH712521	E2	BuPV	Pig	2014-03-05	Australia
NrPV/NYC-D23	KY436034	Genome	LindaV	Pig	2015-01?-?	Austria
Austria2	MZ027894	Genome	LindaV	Pig	2016-?-?	Austria

Supplementary Table S4. Overview of initial alignments tested for recombination and substitution saturation including coverage, number of sequences and sequence length. CR = conserved regions. The conserved regions 1, 2, 3 and 4 correspond to the amino acid positions 189–418, 1547– 2321, 2397–2688 and 3312–3837 numbered according to the reference sequence for BVDV-1 SD-1 (accession number M96751) (Smith *et al.*, 2017). The concatenated regions* covers the 5′UTR, C, Erns, E1 and E2 regions.

Alignment	Data	Coverage	Sequences (n)	Lenght (bp)	Recombination	Saturation
PhoPev alignment	Nucleotides	Concatenated regions*	8	2,502	No → in	No
PhoPev alignment	Nucleotides	E2	8	1,137	No → in	No
PhoPeV, BuPV and LindaV	Nucleotides	E2	19	1,065	No → in	Yes
PhoPeV, BuPV and LindaV	Nucleotides	CR 1	7	687	Yes → out	Yes
PhoPeV, BuPV and LindaV	Nucleotides	CR 2	8	2,325	No → in	Yes
PhoPeV, BuPV and LindaV	Nucleotides	CR 3	8	876	No → in	Yes
PhoPeV, BuPV and LindaV	Nucleotides	CR 4	8	1,575	Yes → out	Yes
PhoPeV, BuPV and LindaV	Nucleotides	CR 2 and 3	10	3,201	No → in	Yes
PhoPeV, BuPV and LindaV	Nucleotides	Concatenated regions*	9	2,004	No → in	Yes
PhoPeV, BuPV and LindaV	Amino acids	Genome	8	3,748		
PhoPeV, BuPV and LindaV	Amino acids	Concatenated	12	710		
PhoPeV, BuPV and LindaV	Amino acids	E2	16	374		
All known pestiviruses	Amino acids	CR 1, 2, 3 and 4	112	1,781		

Supplementary Table S5. Results from the Modeltest-NG indicating the best fit substitution model for the data sets based on AIC and BIC. CR = conserved region.

Alignment	Data	Coverage	Sequences (n)	Modeltest-ng
PhoPev alignment	Nucleotides	Concatenated regions*	8	TrN → TN93
PhoPev alignment	Nucleotides	E2	8	TrN → TN93
PhoPeV, BuPV and LindaV	Nucleotides	CR 2	8	TIM2
PhoPeV, BuPV and LindaV	Nucleotides	CR 3	8	GTR
PhoPeV, BuPV and LindaV	Nucleotides	CR 2 and 3	10	TIM2
PhoPeV, BuPV and LindaV	Amino acids	Genome	8	WAG
PhoPeV, BuPV and LindaV	Amino acids	Concatenated regions*	12	JTT
PhoPeV, BuPV and LindaV	Amino acids	E2	16	JTT
All known pestiviruses	Amino acids	CR 1, 2, 3, 4	112	JTT

Supplementary Table S6. Results of initial BEAST tests of nucleotide and amino acid alignments of PhoPeV, BuPV and LindaV sequences run with a strict clock and 20,000,000 MCMC. Runs assigned with * provided ESS values > 100. Indicating that some priors did not converge under these conditions. The rest of the runs had ESS values > 200.

Alignment	Data	Clock	Tree model	Tree height	Posterior	Likelihood	Prior
E2	Nucleotide	Strict	Bayesian skyline	349 [205, 509]	1593	2725	1080
E2	Nucleotide	Strict	Constant coalescence	199 [142, 266]	2980	3113	1868
E2	Nucleotide	Strict	Exponential coalescence	235 [155, 325]	2412	2533	1546
E2	Amino acids	Strict	Bayesian skyline	250 [115, 433]	418	3422	318
E2	Amino acids	Strict	Constant coalescence	135 [96, 181]	3438	4576	2209
E2	Amino acids	Strict	Exponential coalescence	153 [105, 206]	2733	3787	1802
Concatenated	Amino acids	Strict	Bayesian skyline	358 [158, 634]	1632	4242	1524
Concatenated	Amino acids	Strict	Constant coalescence	171 [119, 226]	5579	4644	4089
Concatenated	Amino acids	Strict	Exponential coalescence	192 [134, 264]	5649	4419	3946
Conserved region 2*	Nucleotide	Strict	Bayesian skyline	463 [174, 862]	854	843	780
Conserved region 2	Nucleotide	Strict	Constant coalescence	194 [139, 259]	1668	1313	1739
Conserved region 2	Nucleotide	Strict	Exponential coalescence	244 [159, 337]	1683	1134	1825
Conserved region 3*	Nucleotide	Strict	Bayesian skyline	1622 [107, 4073]	392	1165	391
Conserved region 3	Nucleotide	Strict	Constant coalescence	134 [85, 191]	3463	2126	2406
Conserved region 3	Nucleotide	Strict	Exponential coalescence	186 [105, 291]	3244	1652	1557
Genome	Amino acids	Strict	Bayesian skyline	1919 [266, 3860]	552	3126	531
Genome	Amino acids	Strict	Constant coalescence	262 [186, 343]	4300	2519	2413
Genome	Amino acids	Strict	Exponential coalescence	326 [209, 453]	4959	3107	2603

Supplementary Table S7. Results of path sampling analyses of the PhoPeV alignment evaluating the best fit model for the data by the calculation of likelihood scores. The analyses were performed under different combinations of clock models (strict clock, relaxed clock lognormal and relaxed clock exponential) and tree prior specifying population dynamics (coalescence constant, coalescence exponential growth, coalescence Bayesian skyline and birth death models). Path sampling analyses were conducted with a chain length of 1,600,000 generations, alpha of 0.3, burn-in percentage of 10, a pre-burn-in of 10 and number of steps set to 112. Tree height estimates correspond to the time to the most recent common ancestor (tMRCA) shared between sequences included in the analysis and were obtained through BEAST v2.6.3 analyses with settings as described in the table and chain length of 200,000,000 Markov Chain Monte Carlo (MCMC). The model with the highest support is marked in bold in the marginal likelihood column.

**Number**	**Clock**	**Tree Model**	**Substitution model**	**TreeHeight**	**Marginal likelihood**
1	Strict	Bayesian skyline	TN93	102 [43, 184]	-4907,43
2	Strict	Constant coalescence	TN93	53 [32, 115]	-4906,68
3	Strict	Exponential coalescence	TN93	54 [39, 71]	-4908,30
4	Lognormal	Bayesian skyline	TN93	26 [13, 68]	-4905,48
5	Lognormal	Constant coalescence	TN93	21 [13, 44]	-4903,56
6	Lognormal	Exponential coalescence	TN93	20 [13, 44]	-4905,39
7	Exponential	Bayesian skyline	TN93	18 [13, 29]	-4905,07
8	Exponential	Constant coalescence	TN93	19 [13, 28]	**-4902,58**
9	Exponential	Exponential coalescence	TN93	18 [13, 29]	-4904,86
13	Strict	BirthDeath	TN93	134 [48, 277]	-4907,59

Supplementary Table S8. Results of path sampling analyses of the PhoPeV, BuPV and LindaV alignment evaluating the best fit model for the data by the calculation of a marginal likelihood (ML) score. The analyses were performed under different combinations of clock models (strict clock, relaxed clock lognormal and relaxed clock exponential) and tree prior specifying population dynamics (coalescence constant (CC), coalescence exponential (EC) growth, coalescence Bayesian skyline (BS) and birth death (BD) model). Path sampling analyses were conducted with a chain length of 1,600,000 M generations, alpha of 0.3, burn-in percentage of 10, a pre-burn-in of 10 and number of steps set to 112. Tree height estimates correspond to the time to the most recent common ancestor (tMRCA) shared between sequences included in the analysis and were obtained through BEAST v2.6.3 analyses with settings as described in the table generated with a chain length of 200,000,000 Markov chain Monte Carlo (MCMC). The model with the highest support is marked with black in the marginal likelihood (ML) column.

Number	Clock	Tree Model	TreeHeight	PhoPeV	BuPeV	Linda	ML
1	Strict	BS	201 [104, 326]	2006 [2002, 2009]	2002 [2000, 2004]	2012 [2009, 2015]	-5152,59
2	Strict	CC	120 [80, 165]	2007 [2006, 2009]	2003 [2002, 2004]	2014 [2012, 2015]	-5160,04
3	Strict	EC	142 [87, 203]	2007 [2005, 2009]	2003 [2002, 2004]	2013 [2011, 2015]	-5154,91
4	Lognormal	BS	61 [13, 174]	2005 [1998, 2009]	1997 [1984, 2004]	2011 [2002, 2015]	-5137,01
5	Lognormal	CC	24 [13, 42]	2008 [2007, 2009]	2002 [1998, 2004]	2013 [2010, 2015]	-5133,50
6	Lognormal	EC	26 [13, 52]	2008 [2007, 2009]	2002 [1998, 2004]	2014 [2010, 2015]	**-5133,43**
7	Exponential	BS	48 [14, 111]	2008 [2007, 2009]	2002 [1997, 2004]	2014 [2010, 2015]	-5139,56
8	Exponential	CC	26 [14, 44]	2009 [2008, 2009]	2003 [2001, 2004]	2014 [2012, 2015]	-5136,64
9	Exponential	EC	31 [14, 56]	2009 [2008, 2009]	2003 [2000, 2004]	2014 [2012, 2015]	-5136,25
10	Strict	BD	220 [105, 367]	2005 [2000, 2008]	2002 [2000, 2004]	2012 [2008, 2015]	-5157,35
11	Lognormal	BD	102 [14, 287]	1990 [1944, 2009]	1982 [1927, 2004]	1997 [1952, 2015]	NA
12	Exponential	BD	52	2007	2001	2012	-5143,12

	[17, 205]	[2003, 2009]	[1993, 2004]	[2005, 2015]
